# Exploring the surface epitope and nuclear localization analysis of *porcine circovirus type 3* capsid protein

**DOI:** 10.1186/s13568-023-01652-6

**Published:** 2023-12-08

**Authors:** Chia-Chun Chang, Ching-Ying Wu, Jhao-Guan Ciou, Ching-Wei Wu, Yi-Chen Wang, Hui-Wen Chang, Maw-Sheng Chien, Chienjin Huang

**Affiliations:** 1 Graduate Institute of Microbiology and Public Health, College of Veterinary Medicine, National Chung Hsing University, 145 Xingda Road, Taichung, 40227 Taiwan; 2 Research Center for Animal Medicine, National Chung Hsing University, 145 Xingda Road, Taichung, 40227 Taiwan; 3 Graduate Institute of Molecular and Comparative Pathobiology, National Taiwan University, 1 Roosevelt Road Section 4, Taipei, 10617 Taiwan; 4 Graduate Institute of Veterinary Pathobiology, College of Veterinary Medicine, National Chung Hsing University, 145 Xingda Road, Taichung, 40227 Taiwan

**Keywords:** *Porcine circovirus 3*, Capsid protein, Monoclonal antibody, Nuclear localization, Cytotoxicity, Virus-like particle

## Abstract

*Porcine circovirus 3* (PCV3) is a newly emerging virus associated with porcine dermatitis and nephropathy syndrome (PDNS) and reproductive disorders, impacting global pig populations. Porcine circoviruses contain two major open reading frames (ORFs), and the ORF2 encodes the viral capsid protein (Cap). Cap is the most antigenic structural protein and an ideal candidate for the development of vaccines and diagnostic reagents. This study generated a monoclonal antibody (MAb) specific to PCV3 Cap, MAb CCC160, for diagnosis and pathogenesis studies of this novel virus. The MAb specifically recognized PCV3-infected swine lymph node tissue in an immunohistochemical analysis confirming its clinical diagnostic potential. In addition, a novel linear B-cell epitope recognized by MAb CCC160 was identified at the amino acid region 120–134 of Cap. Nuclear localization analysis of PCV3 Cap revealed a potential nuclear localization signal (NLS) in the middle region (aa 131–143) in addition to the dominant N-terminal NLS that is already known. A cell viability assay further demonstrated that the cytotoxicity of PCV3 Cap is correlated with its nuclear localization, indicating a crucial role of Cap in the pathogenic mechanism of PCV3. A full-length construct of PCV3 Cap was successfully expressed using a baculovirus expression system and purified recombinant proteins self-assembled into virus-like particles (VLPs). The protein constitution of the VLPs was confirmed by MAb CCC160 recognition, indicating the correct conformation and specificity of VLP and exhibiting the linear epitope aa 120–134 on the VLP surface. These results provide insights for developing diagnostic tools and potential VLP vaccines for PCV3, revealing its pathogenesis and antigenic properties.

**Supplementary Information:**

The online version contains supplementary material available at 10.1186/s13568-023-01652-6.

## Introduction

*Porcine circovirus* (PCV), belonging to the family *Circoviridae* and genus *Circovirus*, is a small nonenveloped swine virus with a circular single-stranded DNA genome (Rosario et al. 2017). PCV1 is a nonpathogenic cell-culture-derived virus discovered in 1974 as a contaminant of a porcine kidney (PK-15) cell line (Tischer et al. 1974). PCV2, identified in 1997, has been considered the main pathogen of PCV-associated diseases (PCVADs), including postweaning multisystemic wasting syndrome, porcine dermatitis and nephropathy syndrome (PDNS), respiratory disease, and reproductive failure (Opriessnig et al. 2007). PCVADs seriously affect pig health and are regarded as some of the most economically important swine diseases worldwide (Segalés 2012). In 2015, a newly emerging virus, *Porcine circovirus 3* (PCV3), was first identified in pigs suffering cardiac and multisystemic inflammation and in sows that died from PDNS (Palinski et al. 2017; Phan et al. 2016). Therefore, it is necessary to re-examine the causes of PCVADs and to investigate the role of PCV3 in them. Since 2016, many countries in America, Asia, and Europe have reported the detection of PCV3 and the virus is associated with clinical diseases in pigs, including respiratory disease, digestive disorders, inflammatory disorders, and reproductive failure (Palinski et al. 2017; Kedkovid et al. 2018). PCV3 has spread worldwide, and poses a potential threat to the health of pigs globally and may cause significant economic losses in the pig industry.

All PCVs are similar in structure. PCV3 has a 2000-bp single-stranded DNA genome with two major open reading frames (ORFs). ORF1 encodes the replication-associated protein (Rep) and ORF2 encodes the capsid protein (Cap) (Ouyang et al. 2019). According to a gene sequence analysis, the similarity between the whole genomic DNAs of PCV3 and PCV2 is < 50%, and the amino acid identities of ORF1 and ORF2 between PCV3 and PCV2 are only 48% and 26–37%, respectively (Palinski et al. 2017; Phan et al. 2016). Therefore, the development of diagnostic reagents to detect and differentiate PCV3 infections is crucial for the control of this newly emerging disease agent.

The Cap protein of PCVs is the only structural protein that assembles into the outer shell of the virus, and is considered the most antigenic protein. The N-terminus of Cap protein is rich in basic amino acids and displays nuclear localization signals (NLSs) as identified in PCV2 Cap (Liu et al. 2001) and PCV3 Cap (Mou et al. 2019). In addition, PCV2 Cap was reported to cause cell death in PK-15 cells but not in HEK293T cells, suggesting that capsid protein is cytotoxic to porcine cells (Walia et al. 2014). Furthermore, NLS deletion of PCV2 Cap resulted in reduced cell death (Yu et al. 2021), suggesting that nuclear localization sequences play an important role in the cytotoxicity of Cap.

PCV3 Cap protein contains 214 amino acid (aa), and the prediction of immune epitopes in the protein identified seven potential epitopes, spanning almost the whole surface of the protein, and indicated that the amino acid at site 24 distinguishes clades PCV3a and PCV3b (Li et al. 2018a). A study showed that the amino acids at sites 10, 24, 27, 77, 104, and 150 are frequently mutated in global strains (Qi et al. 2019). This suggests that the N-terminal half of PCV3 Cap has a tendency to mutate and that the C-terminal half of PCV3 Cap is more likely to be conserved. Therefore, the C-terminal region of PCV3 Cap is more suitable as an antigenic candidate region for the production of diagnostic reagents. Monoclonal antibody (MAb) has become an important tool in molecular research due to their high specificity in targeting certain antigens, and is widely used as a diagnostic laboratory tool to detect the antigens of pathogens (Edwards 1981). In this study, we expressed and purified the C-terminal half of the recombinant PCV3 Cap (PCV3rCap) protein with which to prepare a MAb that specifically recognizes PCV3 Cap. The specificity of this MAb and its potential development as a diagnostic reagent was tested with several serological methods, including western blotting analysis, immunofluorescence assay (IFA), and immunohistochemistry. The subcellular distribution of PCV3 Cap was further analyzed with the MAb specific to the viral protein.

## Materials and methods

### Construction of recombinant plasmids containing defined codon regions of PCV3 ORF2

The sequence of PCV3 strain TW16 (GenBank accession no. MN510467) was obtained from the lymph-node tissues of sick pigs with polymerase chain reaction (PCR), as described previously (Chang et al. 2021). The PCV3 TW16 Cap gene fragment encoding aa 110–214 was codon-optimized and synthesized (GenScript, USA) (Additional file 1: Fig. S1), and cloned into the *Escherichia coli* expression vector pET24a. Two codon-optimized gene fragments encoding aa 110–160 and 160–214 were also amplified with PCR and cloned into pET32a. The PCV2 Cap gene (GenBank accession no. AY885225) was codon-optimized and synthesized (GenScript, USA), and cloned into pET24a. All these constructs contain a C-terminal six-histidine (6-His) tag. The original full-length PCV3 Cap gene was cloned into the mammalian expression vector pcDNA4 for an IFA. A N-terminal NLS deletion mutant (PCV3Cap∆NLS_N_) was constructed by PCR cloning using primer F: 5'-AATCCGAATTCTATGTCAGAAGAAAACTATTCATTAGGAGGCCC-3' and R: 5'-ATCGAGATATCTTAGAGAACGGACTTGTAACGAATCCA-3’. Further mutations at the potential NLS were synthesized (Abclonal, USA), followed by cloned into pcDNA4c to generate the PCV3CapNLS_P_m and PCV3Cap∆NLS_N_NLS_P_m, respectively.

### Expression and purification of PCV3rCap

*Escherichia coli* BL21 cells were transformed with the recombinant plasmids. The cells were induced for protein expression and were harvested as previously described (Wu et al. 2011). The cell pellet was resuspended in denaturation buffer (50 mM sodium phosphate, 300 mM sodium chloride, 6 M urea, 10 mM imidazole, pH 7.4), and centrifuged to remove any debris. The supernatant was filtered through a 0.45 μm filter membrane (Sartorius, Göttingen, Germany) and purified with Ni–NTA agarose (Qiagen, Hilden, Germany) affinity chromatography. The target protein was eluted with elution buffer (50 mM sodium phosphate, 300 mM sodium chloride, 6 M urea, 150 mM imidazole, pH 7.4).

### Preparation of MAbs specific to PCV3 Cap with hybridoma technology

Three 6-week-old female BALB/c mice were purchased from the National Laboratory Animal Center (Taipei, Taiwan), and were immunized by intraperitoneal injection with 30 μg of purified recombinant protein PCV3rCap110–214 mixed with an equal volume of Freund’s complete adjuvant (Sigma-Aldrich, St. Louis, MO, USA). After 2 weeks, the mouse received a booster injection of 30 μg purified PCV3rCap110–214 mixed with an equal volume of Freund’s incomplete adjuvant (Sigma). Immunized mice received a final intraperitoneal booster of 30 μg purified protein without adjuvant. Mice were sacrificed,and spleen cells fusion with NS-1 myeloma cell was carried out as previously described (Wu et al. 2011). The subclass of the MAb was determined with the Pierce Rapid ELISA Mouse mAb Isotyping Kit (Thermo Fisher Scientific). All mouse experimental procedures were reviewed and approved by the Institutional Animal Care and Use Committee (IACUC) of National Chung Hsing University (Taichung, Taiwan) under IACUC approval number 108–131.

### Enzyme-linked immunosorbent assay (ELISA) and competitive ELISA

A 96-well EIA Assay Microplate (Corning, Corning, NY, USA) was coated with 50 μL of diluted PCV3rCap110–214 and incubated overnight at 4 °C. The plate was then washed with PBS (phosphate-buffered saline) containing 0.05% Tween 20 (PBST) and blocked with PBS containing 3% bovine serum albumin (BSA) for 1 h at 37 °C. The culture supernatants of the hybridoma cells were added to the wells and incubated overnight at 4 °C. Mouse immune serum and preimmune serum (1:1,000) were used as the positive and negative controls, respectively. For competitive ELISA, the defined monoclonal antibody (MAb) was incubated with each synthetic peptide (1 μg/μL) at 37 °C for 1 h, followed by addition to the wells and overnight incubation at 4 °C. After the cells were washed with PBST, a horseradish peroxidase (HRP)-conjugated goat anti-mouse IgG antibody (Jackson ImmunoResearch Laboratories, Inc., West Grove, PA, USA), diluted 5,000-fold in PBS containing 1.5% BSA, was added and the cells were incubated for 1 h at 37 °C. After washing the plate with PBST and the 3,3′, 5, 5′-tetramethylbenzidine (TMB) ELISA substrate (Invitrogen, Waltham, MA, USA) was added. The plate was incubated in the dark at room temperature for 10 min, and then stop solution (1 N HCl) was added. The OD_450_ was measured with the MRX Revelation Microplate Reader (Dynex, Chantilly, VA, USA).

### Sodium dodecyl sulfate (SDS)-polyacrylamide gel electrophoresis (PAGE) and western blotting analysis

Protein electrophoresis and western blotting analysis were performed as previously described (Wu et al. 2012). The culture supernatant of hybridoma cell secreting monoclonal antibody CCC160 was added as primary antibody and incubated overnight at 4 °C. The anti-His antibody (Bio-Rad) at 1:1000 dilution was used as control. The membrane was soaked in TMB substrate solution (Invitrogen) for color development.

### Cell line

Porcine alveolar macrophage (PAM) cells and swine testis (ST) cells were cultured in DMEM (Gibco) medium with 10% fetal bovine serum (FBS) (Gibco) at 37 °C with 5% CO_2_ incubator. *Spodoptera frugiperda* (Sf9) insect cells were cultured in ExpiSf™ CD medium (Thermo Fisher Scientific) at 27 °C incubator.

### Transfection and immunofluorescence assay (IFA)

The transfection was performed using X-tremeGENE™ HP DNA Transfection Reagent (Roche, Basel, Switzerland) according to the manufacturer protocol. PAM cells were seeded in 24 well plates at 70% confluence, then transfected with 0.5 µg of recombinant plasmid pcDNA4cPCV3Cap DNA. ST cells were seeded in 8 well slides at 50% confluence, then transfected with 0.3 µg of various pcDNA4c recombinant plasmids expressing defined PCV3 Cap recombinant protein. Cells were incubated for 48 h and then fixed with 4% formaldehyde (Thermo Fisher Scientific) for 10 min. Each MAb solution was added to the wells and the samples incubated overnight at 4 °C. After washing the cells with PBS, AlexaFluor-488-labeled goat anti-mouse IgG antibody (1:800, ThermoFisher Scientific) was added and incubated for 1 h at 37 °C. The plate was washed with PBS and the cells were counterstained with 4,6-diamidino-2-phenylindole (DAPI; SouthernBiotech, Birmingham, AL, USA). The cells were observed under an inverted fluorescence microscope (Olympus IX73, Tokyo, Japan) and a confocal laser-scanning microscope (Olympus FV3000, Tokyo, Japan).

### Immunohistochemistry (IHC)

Paraffin-embedded swine lymph node tissue sections were stained with hematoxylin and eosin (H&E) with the Dako REAL™ EnVision™ Detection System (Dako, Santa Clara, CA, USA) or immunohistochemically stained with the MAb specific to PCV3 Cap (MAb CCC160) or a commercial MAb specific to PCV2 Cap (MAb 36A9, Ingenasa, Madrid, Spain). The 3,3′-diaminobenzidine (DAB) solution (Bond Biotech, Parkville, MO, USA) was added as the substrate to detect positive results as a dark brown insoluble product.

### Cell viability assay

ST cells (60% confluence) in a 96-well plate were transfected with 0.3 μg of various pcDNA4c recombinant plasmids expressing defined PCV3 Cap recombinant protein. After 48 h of transfection, cell viability was assessed by adding 10 μL of WST-8 reagent (AAT Bioquest, Pleasanton, CA, USA) to each well of the plate and incubating at 37 °C for 1 h. Absorbance was measured at 460 nm using Spark® multimode microplate reader (TECAN, Hamburg, Germany). The experiments were conducted in triplicate.

### Generation of recombinant baculoviruses

Full-length of PCV3 Cap gene with mutation at a potential NLS (R133A, K140A, and R143A) was codon optimized and synthesized by ABclonal (Woburn, MA, USA) according to codon usage bias of *Spodoptera frugiperda* (Additional file 1: Fig. S1). Subsequently, it was cloned into the pFastBac1 expression vector (Thermo Fisher Scientific, USA) and transformed into DH10Bac™ competent cells (Thermo Fisher Scientific) to generate the recombinant Bacmid. Sf9 cells were seeded at a density of 2.5 × 10^6^ cells/mL in a 125 mL vented shake flask, followed by transfection with the recombinant Bacmid using ExpiFectamine™ Sf Transfection Reagent (Thermo Fisher Scientific). The recombinant baculoviruses Bac/PCV3Cap were harvested from the culture medium and stored at − 70 °C.

### Expression and purification of PCV3 virus-like particle

Sf9 cells were seeded in a 500 mL vented shake flask at a density of 5 × 10^6^ cells/mL with the addition of ExpiSf™ enhancer (Thermo Fisher Scientific), and cultured for 18 h followed by infection with 1 mL of recombinant baculoviruses. At 96 h post-infection, Sf9 cells were harvested by centrifugation, resuspended with 10 mL of equilibration buffer (20 mM phosphate buffer and 500 mM NaCl, pH 7.4), and then sonicated on ice for 10 cycles of 10 s pulses at 60 s intervals using a Vibra-Cell Ultrasonic Processors VCX750 (Sonics & Material, Newtowns, USA) at 25% amplitude. The supernatant was collected by centrifugation and filtered through a 0.22 μm filter, and further purified by ÄKTA Fast protein liquid chromatography (FPLC) system (GE-Healthcare Life Sciences, USA).

### Transmission electron microscopy

A carbon-coated cooper grid was coated with 10 µl of purified PCV3 VLP for 5 min at room temperature, following by wash with distilled deionized water (DDW) for 10 s. Subsequently, the grid was stained with 1% phosphotungstic acid (PTA) for 8 min and residual dye was removed using filter paper. For Immunogold labeling, samples were coated at a carbon-coated nickel grid for 5 min at room temperature, followed by washing with PBS and blocking with 1% BSA in PBS. The grid was then incubated with the MAb specific to PCV3 Cap at 4 °C overnight. After washing with 1% BSA in PBS, goat anti-mouse antibodies conjugated with 15 nm gold particles (SIGMA) were added and incubated at 37 °C for 1 h. Subsequently, the grids were washed twice with 0.1% BSA in PBS and then rinsed with PBS four times. The grids were then fixed with 1% glutaraldehyde for 5 min followed by two washes with PBS, and six times rinses with DDW. Finally, the grids were stained with 1% PTA for 8 min, and then observed under a transmission electron microscope (TEM) (JEMI 1400, JEOL, Tokyo, Japan).

## Results

### Preparation and characterization of MAbs specific to PCV3 Cap

**Fig. 1:**
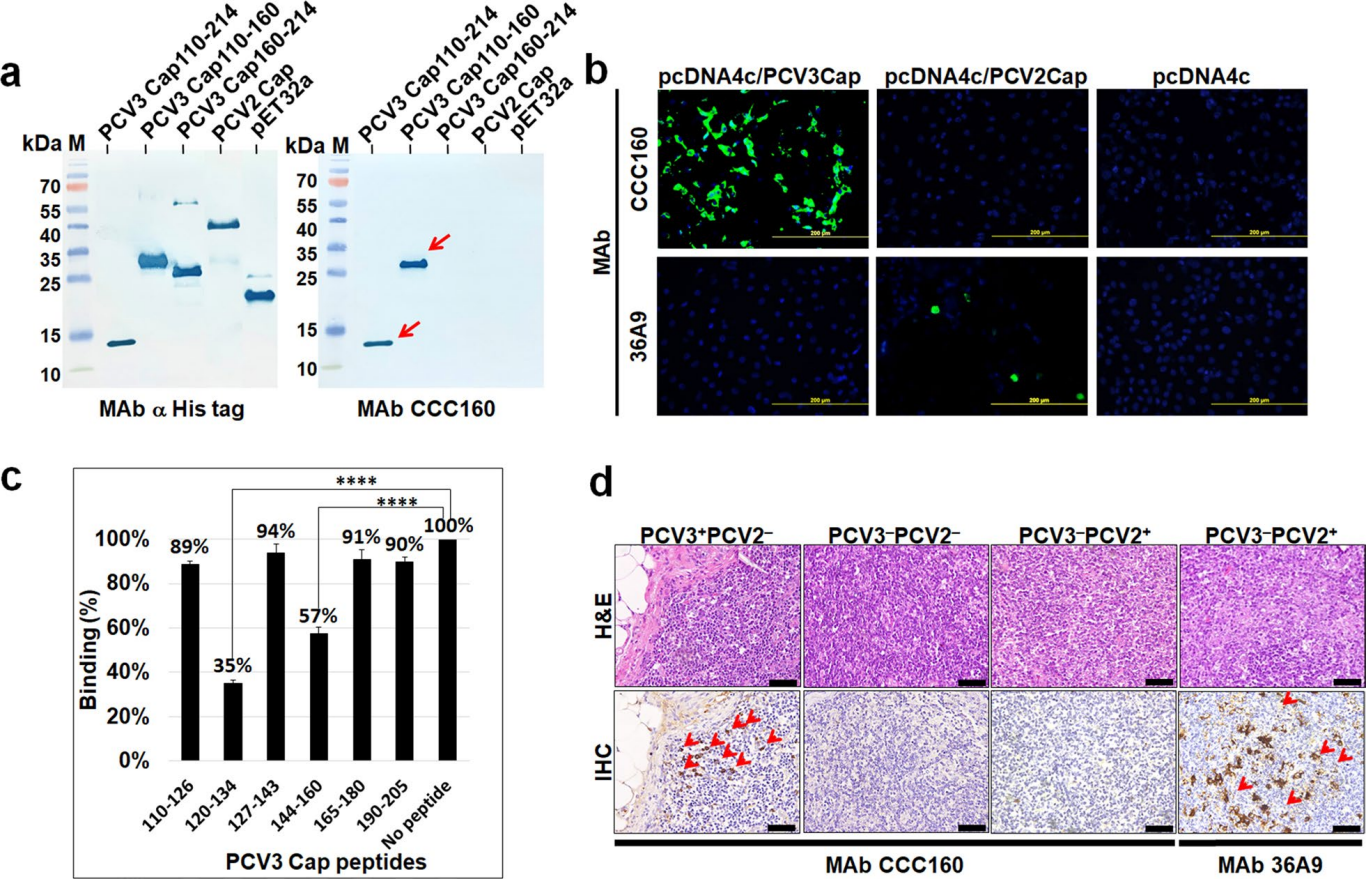
Characterization of the monoclonal antibody specific to PCV3 Cap. **a** The MAb CCC160 was examined by western blotting analysis for its recognition of various purified recombinant proteins, including three recombinant PCV3 Cap proteins (Cap110–214, Cap110–160, and Cap161–214), a recombinant PCV2 Cap protein, and the pET32a carrier protein. Lane M: marker. **b** ST cells were transfected with pcDNA4c/PCV3 Cap and pcDNA4c/PCV2 Cap, respectively, and fixed at 48 h post-transfection, followed by immunofluorescence assay with the MAb CCC160 or the commercial MAb 36A9 which is specific to PCV2 Cap. **c** Epitope mapping of MAb CCC160 was performed using a competitive ELISA. The results were presented as the mean ± SD of triplicate experiments. Statistical significance was assessed with one-way ANOVA. **** P-value ≤ 0.0001 when compared to the cell control group. **d** Characterization of the specificity of MAb CCC160 with immunohistochemistry (IHC). H&E staining of lymph node tissues from PCV3^+^PCV2^−^ pigs and PCV3^−^PCV2^+^ pigs showed macrophage proliferation and mild lymphocyte depletion, whereas that from PCV3^−^PCV2^−^ pigs showed normal morphology. The IHC staining results showed that MAb CCC160 specifically recognized PCV3-infected tissues and a positive signal was observed (arrowed) but was not observed in normal or PCV2-infected tissues. The MAb 36A9 was used as a control to recognize PCV2-infected tissue

The defined region spanning aa residues 110–214 of the PCV3rCap protein was expressed in the *E. coli* pET24a system. PCV3rCap110–214, expressed with a six-histidine (6-His) C-terminal tag, was purified with Ni–NTA affinity chromatography and then used as the antigen to immunize BALB/c mice for the production of hybridomas. A stable hybridoma secreting antibody specific to PCV3rCap was selected, cloned, and designated MAb CCC160, which belongs to the IgG2a subclass. The specificity of MAb CCC160 was analyzed with western blotting using a variety of *E. coli-*expressed recombinant PCV3rCap and PCV2rCap proteins. As shown in Fig. 1a, MAb CCC160 specifically recognized the PCV3rCap110–214 and PCV3rCap110–160, but not PCV3rCap160–214 or PCV2rCap. In addition, MAb CCC160 was able to recognize PAM cells transfected with pcDNA4c/PCV3Cap and showed that the PCV3 Cap protein mainly accumulated in the cell nucleus with a minor presence in the cytoplasm (Fig. 1b). In contrast, no signal was observed in PAM cells transfected with pcDNA4c/PCV2Cap, which were recognized by the commercial MAb 36A9 specific to PCV2 Cap. Western blotting results showed that the epitope was located at aa 110–160, and competitive ELISA was used for further fine mapping of the MAb CCC160 epitope. Potential B cell epitopes of PCV3 Cap110–214 were predicted by Immune Epitope Database (IEDB) Analysis Resource (Vita et al. 2019) and six peptides were synthesized (Allbio, Taiwan). The binding affinity of MAb CCC160 to PCV3rCap110–214 was remarkably reduced to 35% when incubated with the synthetic peptide 120–134, and moderately decreased to 57% when incubated with the peptide 144–160 (Fig. 1c). Furthermore, clinical lymph node specimens from PCV3^+^PCV2^−^, PCV3^−^PCV2^+^, and PCV3^−^PCV2^−^ pigs, confirmed with PCR, were sectioned for immunohistochemical staining with MAb CCC160. As shown in Fig. 1d, positive signals for PCV3 antigen were observed in the mononuclear cells from the PCV3^+^PCV2^−^ pigs, whereas no signal was present in the samples from PCV3^−^PCV2^+^ or PCV3^−^PCV2^−^ pigs. Therefore, MAb CCC160 specifically recognized PCV3-infected tissues and differentiated them from PCV2-infected tissues, which were stained with a commercial MAb directed against PCV2 Cap (Fig. 1d).

### Nuclear localization analysis of PCV3 Cap

**Fig. 2:**
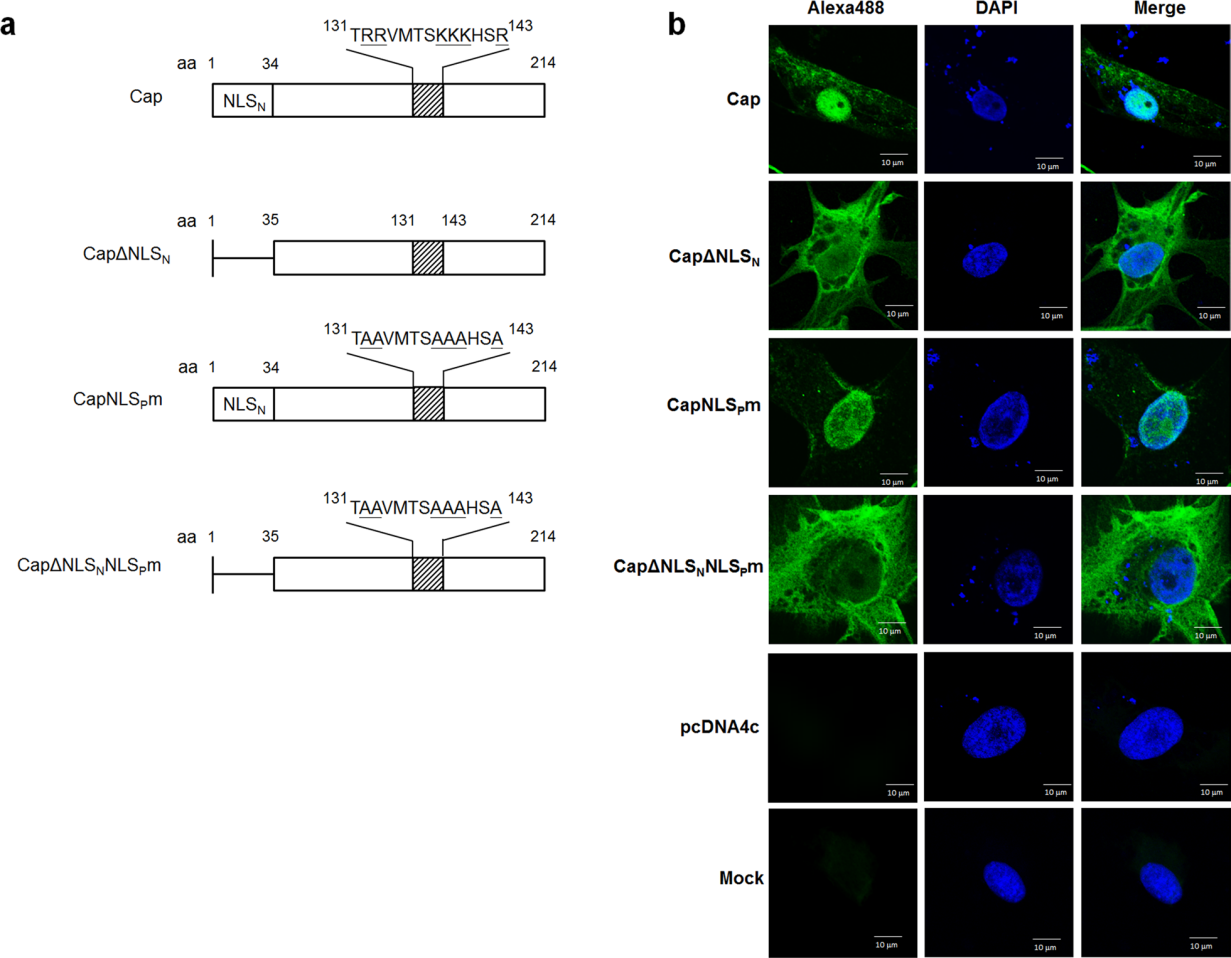
Analysis of PCV3 Cap protein subcellular distribution by immunofluorescence assay. **a** Schematic diagrams of PCV3 Cap mutants. **b** ST cells were transfected with defined recombinant plasmid and fixed at 48 h post-transfection followed by staining with MAb CCC160

**Fig. 3:**
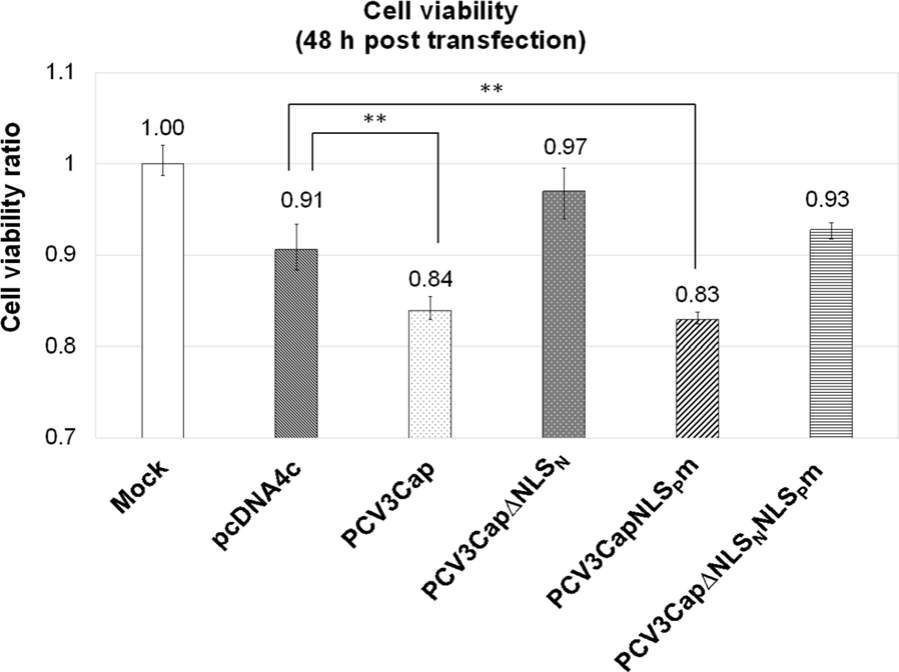
Relative cell viabilities of PCV3 Cap recombinant plasmids transfected-ST cells ST cells were transfected with pcDNA4c/PCV3Cap, pcDNA4c/PCV3Cap∆NLS_N_, pcDNA4c/PCV3CapNLS_P_m, and pcDNA4c/PCV3Cap∆NLS_N_NLS_P_m, respectively. The results were presented as the mean ± SD of triplicate experiments. Statistical significance was assessed with one-way ANOVA. ** P-value ≤ 0.01 when compared to the cell control group

Full length (Cap) and the N-terminal NLS deletion mutant (Cap△NLS_N_) of PCV3 Cap were constructed for nuclear localization analysis with MAb CCC160 by IFA assay (Fig. 2). PCV3 Cap was predominantly accumulated in nuclei of swine testis cells at 48 h post transfection, however, the Cap△NLS_N_ showed distribution in both the cytoplasm and the nuclei (Fig. 2b). Sequence analysis of PCV3 Cap with NLStradamus prediction which is a nuclear localization signals (NLSs) predictor (Nguyen et al. 2009) revealed a potential NLS designated as NLS_P_ at aa 131–143 (TRRVMTSKKKHSR). This region is a short stretch rich in the basic amino acids arginine (R) and lysine (K). To further estimate the potential NLS_P_ activity, two NLS_P_ mutants (NLS_P_m) were constructed by replacing the basic amino acids with alanine (A), as shown in Fig. 2a. PCV3 Cap with NLS_P_ mutation (NLS_P_m) was distributed in nuclei, in contrast, Cap with both the NLS_N_ deletion and NLS_P_ mutation (Cap∆NLS_N_NLS_P_m) exhibited predominant localization in the cytoplasm, as shown in Fig. 2b. In addition, the cytotoxicities of PCV3 Cap and its various mutants were assessed by analyzing cell viability using a cell viability assay. As depicted in Fig. 3, the presence of PCV3 Cap and CapNLS_P_m in the cell nuclei led to a significant reduction in cell viability at 48 h post transfection, while no difference was observed in Cap∆NLS_N_ and Cap∆NLS_N_NLS_P_m.

### Expression and characterization of PCV3 VLP

**Fig. 4:**
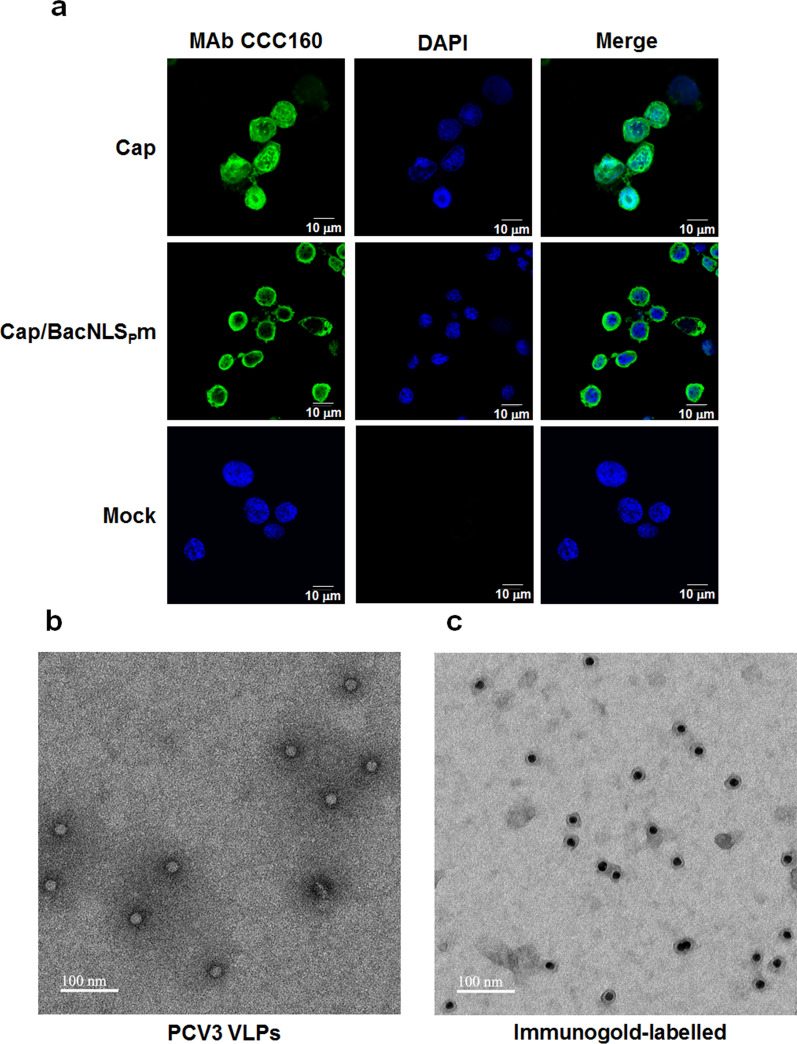
Preparation and characterization of PCV3 Cap VLPs. **a** Expression of full-length PCV3 Cap with mutated NLS_P_ (Cap/BacNLS_P_m) recombinant protein in Sf9 cells. Sf9 cells were infected with the recombinant baculovirus and fixed at 48 h post-infection, followed by immunofluorescence assay with the MAb CCC160. **b** The purified Cap/BacNLS_P_m VLPs were negatively stained (**b**) and further recognized with the MAb CCC160 by immunogold staining (**c**), followed by observation using a TEM. The scale bar is 100 nm

The codon optimized full-length of the PCV3 Cap gene was synthesized into pFastBac1 donor vector to construct the recombinant baculovirus Bac/PCV3Cap. Sf9 cells were infected with recombinant baculovirus for 48 h, expression of PCV3 Cap recombinant proteins was characterized by IFA with MAb CCC160. As shown in Fig. 4a, full-length Cap recombinant proteins were efficiently expressed in Sf9 cells, while Cap/BacNLS_P_m with mutated NLS_P_ (R133A, K140A, and R143A) was mainly distributed in the cytoplasm instead of nuclear accumulation. Purified PCV3 Cap/BacNLS_P_m recombinant proteins were subjected to TEM and immunogold staining. As shown in Fig. 4b, expressed Cap/BacNLS_P_m self-assembled to form VLPs with a size of approximately 20 nm. VLPs were further characterized by immunogold staining with the MAb CCC160 (Fig. 4c).

## Discussion

Since it was first reported, PCV2 has affected pig herds worldwide for more than three decades. Although many commercial vaccines are currently available for PCV2, PCV2-caused PCVADs are still endemic, resulting in serious economic losses in the pig industry (Afghah et al. 2017). In 2015, novel PCV3 was identified, which was associated with PDNS, cardiac and multisystemic inflammation, respiratory disease, and reproductive failure (Palinski et al. 2017; Phan et al. 2016). Several reports have shown that PCV2 and PCV3 frequently coinfected swine herds, with coinfection rates of 3–57% (Chang et al. 2021; Wang et al. 2020). The etiology of PCVADs is becoming more complicated with the further investigation of PCV3. Both PCV2 and PCV3 are clinically associated with PDNS-like diseases, reproductive disorders, and respiratory diseases (Palinski et al. 2017; Kedkovid et al. 2018). The nucleic acids of PCV3 can be detected in the organs, feces, semen and colostrum of infected animals (Tan et al. 2021). Lymphoid tissue lesions are characteristic of PCV2 infection, and PCV3 also causes lymphatic lesions (Palinski et al. 2017; Chianini et al. 2003). Therefore, a diagnostic tool that can specifically detect and differentiate PCV2 and PCV3 infections is urgently required.

Monoclonal antibodies have been widely used in viral research and diagnosis, and a highly specific MAb can be used to differentiate infections if the pathogen appears as a unique subtype, defined by antigenic differences (Siddiqui 2010). Recently, several MAbs directed against the PCV3 Cap protein have been produced. One MAb has been produced that recognized *E. coli*-expressed PCV3rCap in a western blotting analysis and demonstrated its reactivity against PCV3-infected pig tissues with immunohistochemical staining (Li et al. 2018b). An *E. coli*-expressed N-terminally truncated PCV3 Cap was used as the antigen to generate a panel of MAbs for epitope mapping with peptide ELISA, and three conserved linear B-cell epitopes were identified at aa 57–61, aa 140–146, and aa 161–166 (Jiang et al. 2020). In the present study, the MAb CCC160 specific to PCV3 Cap was generated and shown to specifically recognize PAM-expressed PCV3rCap with IFA (Fig. 1b). MAb CCC160 was also shown to specifically recognize the lymph-node tissues of PCV3^**+**^PCV2^−^ pigs, but not in those of PCV3^−^PCV2^−^ or PCV3^−^PCV2^**+**^ pigs, with immunohistochemical staining (Fig. 1d), suggesting its potential utility as a diagnostic reagent for the detection and differentiation of PCV3 infections. For fine epitope mapping, we utilized overlapping synthetic peptides in a competitive ELISA. The results demonstrated that the binding ability of MAb CCC160 to PCV3rCap110–214 was significantly diminished when incubated with peptide aa 120–134 (Fig. 1c). This finding suggests that the epitope recognized by MAb CCC160 is likely located within this region. However, the peptide aa 144–160 also showed moderate inhibition of MAb CCC160 binding activity to Cap. The 3D model constructed based on PCV3 Cap110–214 by SWISS-MODEL (Waterhouse et al. 2018) showed the structures of both peptides were a loop and located adjacent to each other in the tertiary structure (data not shown). Therefore, peptide aa 144–160 may bind MAb CCC160 due to its structural similarity to aa 120–134, resulting in a reduced ability of MAb CCC160 to bind PCV3rCap110–214. Epitope mapping of MAb CCC160 revealed a novel linear B-cell epitope located at aa 120–134, distinct from previously identified antigenic epitopes.

PCV3 Cap predominantly accumulates in the nucleus and its nuclear localization sequence (NLS) has been defined at aa 8–32 (Mou et al. 2019). In the present study, the PCV3 Cap recombinant protein with N-terminal NLS deletion (PCV3Cap∆NLS_N_) showed distribution in the cytoplasm and nucleus (Fig. 3b), suggesting the presence of additional NLS or related sequences that have not been characterized yet. A potential NLS at aa 131–143 (TRRVMTSKKKHSR) was predicted by NLStradamus. However, in the mutant (PCV3CapNLS_P_m) where the basic amino acids within this potential NLS (NLS_P_) were mutated to abolish NLS activity, the protein remained localized within the nucleus. The mutant (PCV3Cap∆NLS_N_NLS_P_m) with both N-terminal NLS deletion and potential NLS mutation demonstrated predominant cytoplasmic distribution. These results indicate that the N-terminal NLS (aa 1–35) of PCV3 Cap plays a major role in nuclear localization, exhibiting competent NLS activity, whereas the potential NLS at aa 131–143 appears to be a minor NLS. The weak nuclear localization activity of NLS_P_ is probably due to its atypical amino acid sequence compared to the consensus sequence K(K/R)X(K/R) of classical monopartite NLS (Kosugi et al. 2009), resulting in lower affinity for binding with the nuclear import protein importin α. In addition, the effect of PCV3 Cap cellular distribution on porcine cell was further analyzed by cell viability assay with WST-8 reagent. As shown in Fig. 3, expression of PCV3 Cap or Cap with NLS_P_ mutation demonstrated significant decreases in cell viabilities, suggesting that accumulation of PCV3 Cap in the cell nucleus may cause severe cytotoxicity, leading to cell death. This result is similar to a previous study which demonstrated that PCV2 Cap could induce cell death in PK-15 cells, and this effect was reversed in the truncated NLS Cap mutant (Walia et al. 2014). PCV3 Cap exhibits toxicity towards porcine cells, and its cytotoxic effect is correlated with the nuclear localization of the protein (Fig. 3). Further research is needed to investigate the cytotoxicity mechanism induced by PCV3 Cap.

Vaccination has been used widely to effectively prevent viral infections in swine. The capsid protein of PCV is considered to be the most immunogenic, making it the optimal candidate for developing PCV3 vaccines. Due to the presence of decoy epitopes in PCV2, it can lead to the generation of non-neutralizing antibodies induced by capsid monomer subunit vaccines (Trible et al. 2012). Subunit vaccines in the form of VLP avoid the exposure of decoy epitopes, thus reducing the production of non-neutralizing antibodies and inducing specific neutralizing antibodies, thereby enhancing vaccine efficacy (Jin et al. 2018). Although it is unclear whether PCV3 possesses such a decoy epitope, VLP mimics the original structure of virus particle and presents viral proteins in a native tertiary conformation, allowing for inducing a highly specific and strong B-cell immune response (Roldão et al. 2010). Therefore, we established a baculovirus expression system to produce full-length of PCV3 Cap. The mutant Cap/BacNLS_P_m with NLS_P_ mutation (R133A, K140A, and R143A) was constructed for relieving nuclear localization that could be a better vaccine candidate. Both wild type and the NLS_p_ mutant could be recognized by MAb CCC160, and Cap/BacNLS_P_m is mainly distributed in the cytoplasm (Fig. 4a), suggesting that those basic amino acids at positions 133, 140, and 143 in NLS_P_ are critical for the nuclear localization of the Cap protein in Sf9 cells. In vaccine development, mutation of PCV3 Cap NLS_P_ reduces nuclear accumulation and cytotoxic effect of the protein, facilitating purification and enhancing production of the recombinant protein. The expressed PCV3 Cap/BacNLS_P_m recombinant proteins self-assembled to form VLPs with correct conformation (Fig. 4b) and specificity (Fig. 4c), indicating that the epitope 120–134 recognized by MAb CCC160 is located on the surface of the VLP. The efficacy of the PCV3 Cap VLP vaccine needs further studies.

In summary, newly emerging PCV3 has spread worldwide, significantly affecting pig health. For accurate detection and the in-depth pathogenesis study of the virus, a MAb specific to PCV3 Cap was generated and confirmed to specifically recognize swine-macrophage-expressed PCV3 Cap protein. MAb CCC160 also highly specifically recognized PCV3-infected pig tissue, suggesting it may have great potential use in clinical diagnosis. The nuclear localization analysis of PCV3 Cap revealed a potential NLS (NLS_P_) in addition to the dominant N-terminal NLS. Cellular distribution of PCV3 Cap is associated with NLSs, and nuclear accumulation of this protein could induce cytotoxic effect in porcine cells. The full-length Cap recombinant proteins with NLS_P_ mutation could form VLP with correct conformation and specificity recognized by MAb CCC160, confirming that the newly identified epitope 120–134 is on the surface of the virion. These findings offer valuable insights into the characteristics and detection of PCV3 and provide significant information regarding the function of PCV3 Cap in viral pathogenicity.

### Supplementary Information

**Additional file 1: Fig S1** PCV3 Cap gene amino acid and nucleotide sequences.

## Data Availability

The data generated during the study are included in this article.

## Abbreviations

PCV: Porcine circovirus
PDNS: Porcine dermatitis and nephropathy syndrome
PCVAD: PCV-associated diseases
ORFs: Open reading frames
Cap: Capsid protein
NLS: Nuclear localization signal
VLP: Virus-like particle
MAb: Monoclonal antibody
IFA: Immunofluorescence assay
IHC: Immunohistochemistry
PCR: Polymerase chain reaction
aa: Amino acids
ELISA: Enzyme-linked immunosorbent assay
PBS: Phosphate-buffered saline
BSA: Bovine serum albumin
SDS: Sodium dodecyl sulfate
PAGE: Polyacrylamide gel electrophoresis
PAM: Porcine alveolar macrophage
ST: Swine testis
Sf9: Spodoptera frugiperda
FBS: Fetal bovine serum
FPLC: Fast protein liquid chromatography
DDW: Distilled deionized water
PTA: Phosphotungstic acid
TEM: Transmission electron microscop

## References

[CR1] Afghah Z, Webb B, Meng XJ, Ramamoorthy S (2017). Ten years of PCV2 vaccines and vaccination: Is eradication a possibility?. Vet Microbiol.

[CR2] Chang CC, Wu CW, Chang YC, Wu CY, Chien M-S, Huang C (2021). Detection and phylogenetic analysis of porcine circovirus type 3 in Taiwan. Arch Virol.

[CR3] Chianini F, Majo N, Segalés J, Domınguez J, Domingo M (2003). Immunohistochemical characterisation of PCV2 associate lesions in lymphoid and non-lymphoid tissues of pigs with natural postweaning multisystemic wasting syndrome (PMWS). Vet Immunol Immunopathol.

[CR4] Edwards PA (1981). Some properties and applications of monoclonal antibodies. Biochem J.

[CR5] Jiang M, Guo J, Zhang G, Jin Q, Liu Y, Jia R, Wang A (2020). Fine mapping of linear B cell epitopes on capsid protein of porcine circovirus 3. Appl Microbiol Biotechnol.

[CR6] Jin J, Park C, Cho SH, Chung J (2018). The level of decoy epitope in PCV2 vaccine affects the neutralizing activity of sera in the immunized animals. Biochem Biophys Res Commun.

[CR7] Kedkovid R, Woonwong Y, Arunorat J, Sirisereewan C, Sangpratum N, Lumyai M, Kesdangsakonwut S, Teankum K, Jittimanee S, Thanawongnuwech R (2018). Porcine circovirus type 3 (PCV3) infection in grower pigs from a Thai farm suffering from porcine respiratory disease complex (PRDC). Vet Microbiol.

[CR8] Kosugi S, Hasebe M, Matsumura N, Takashima H, Miyamoto-Sato E, Tomita M, Yanagawa H (2009). Six classes of nuclear localization signals specific to different binding grooves of importin α. J Biol Chem.

[CR9] Li G, He W, Zhu H, Bi Y, Wang R, Xing G, Zhang C, Zhou J, Yuen KY, Gao GF, Su S (2018). Origin, genetic diversity, and evolutionary dynamics of novel porcine circovirus 3. Adv Sci.

[CR10] Li X, Bai Y, Zhang H, Zheng D, Wang T, Wang Y, Deng J, Sun Z, Tian K (2018). Production of a monoclonal antibody against Porcine circovirus type 3 cap protein. J Virol Methods.

[CR11] Liu Q, Tikoo SK, Babiuk LA (2001). Nuclear localization of the ORF2 protein encoded by porcine circovirus type 2. Virol.

[CR12] Mou C, Wang M, Pan S, Chen Z (2019). Identification of nuclear localization signals in the ORF2 protein of porcine circovirus type 3. Viruses.

[CR13] Nguyen Ba AN, Pogoutse A, Provart N, Moses AM (2009). NLStradamus: a simple Hidden Markov Model for nuclear localization signal prediction. BMC Bioinform.

[CR14] Opriessnig T, Meng XJ, Halbur PG (2007). Porcine circovirus type 2–associated disease: update on current terminology, clinical manifestations, pathogenesis, diagnosis, and intervention strategies. J Vet Diagn Invest.

[CR15] Ouyang T, Niu G, Liu X, Zhang X, Zhang Y, Ren L (2019). Recent progress on porcine circovirus type 3. Infect Genet Evol.

[CR16] Palinski R, Piñeyro P, Shang P, Yuan F, Guo R, Fang Y, Byers E, Hause BM (2017). A novel porcine circovirus distantly related to known circoviruses is associated with porcine dermatitis and nephropathy syndrome and reproductive failure. J Virol.

[CR17] Phan TG, Giannitti F, Rossow S, Marthaler D, Knutson TP, Li L, Deng X, Resende T, Vannucci F, Delwart E (2016). Detection of a novel circovirus PCV3 in pigs with cardiac and multi-systemic inflammation. Virol J.

[CR18] Qi S, Su M, Guo D, Li C, Wei S, Feng L, Sun D (2019). Molecular detection and phylogenetic analysis of porcine circovirus type 3 in 21 provinces of China during 2015–2017. Transbound Emerg Dis.

[CR19] Roldão A, Mellado MCM, Castilho LR, Carrondo MJ, Alves PM (2010). Virus-like particles in vaccine development. Expert Rev Vaccines.

[CR20] Rosario K, Breitbart M, Harrach B, Segalés J, Delwart E, Biagini P, Varsani A (2017). Revisiting the taxonomy of the family Circoviridae: establishment of the genus Cyclovirus and removal of the genus Gyrovirus. Arch Virol.

[CR21] Segalés J (2012). Porcine circovirus type 2 (PCV2) infections: clinical signs, pathology and laboratory diagnosis. Virus Res.

[CR22] Siddiqui M (2010). Monoclonal antibodies as diagnostics; an appraisal. Indian J Pharm Sci.

[CR23] Tan CY, Lin CN, Ooi PT (2021). What do we know about porcine circovirus 3 (PCV3) diagnosis so far?: a review. Transbound Emerg Dis.

[CR24] Tischer I, Rasch R, Tochtermann G (1974). Characterization of papovavirus and picornavirus-like particles in permanent pig kidney cell lines. Zent Bakteriol Orig A.

[CR25] Trible BR, Suddith AW, Kerrigan MA, Cino-Ozuna AG, Hesse RA, Rowland RR (2012). Recognition of the different structural forms of the capsid protein determines the outcome following infection with porcine circovirus type 2. J Virol.

[CR26] Vita R, Mahajan S, Overton JA, Dhanda SK, Martini S, Cantrell JR, Wheeler DK, Sette A, Peters B (2019). The immune epitope database (IEDB): 2018 update. Nucleic Acids Res.

[CR27] Walia R, Dardari R, Chaiyakul M, Czub M (2014). Porcine circovirus-2 capsid protein induces cell death in PK15 cells. Virol.

[CR28] Wang Y, Noll L, Lu N, Porter E, Stoy C, Zheng W, Liu X, Peddireddi L, BaiJ NM (2020). Genetic diversity and prevalence of porcine circovirus type 3 (PCV3) and type 2 (PCV2) in the Midwest of the USA during 2016–2018. Transbound Emerg Dis.

[CR29] Waterhouse A, Bertoni M, Bienert S, Studer G, Tauriello G, Gumienny R, Heer FT, de Beer TAP, Rempfer C, Bordoli L, Lepore R, Schwede T (2018). SWISS-MODEL: homology modelling of protein structures and complexes. Nucleic Acids Res.

[CR30] Wu CW, Chien MS, Liu TY, Lin GJ, Lee WC, Huang C (2011). Characterization of the monoclonal antibody against classical swine fever virus glycoprotein Erns and its application to an indirect sandwich ELISA. Appl Microbiol Biotechnol.

[CR31] Wu PC, Lin WL, Wu CM, Chi JN, Chien MS, Huang C (2012). Characterization of porcine circovirus type 2 (PCV2) capsid particle assembly and its application to virus-like particle vaccine development. Appl Microbiol Biotechnol.

[CR32] Yu W, Sun Y, He Q, Sun C, Dong T, Zhang L, Zhan Y, Wang N, Yang Y, Sun Y (2021). Mitochondrial localization signal of porcine circovirus type 2 capsid protein plays a critical role in cap-induced apoptosis. Vet Sci.

