# Supplementary figures and images for: Exploring the surface epitope and nuclear localization analysis of porcine circovirus type 3 capsid protein
Source: AMB Express. 2023 Dec 8;13:141. doi: 10.1186/s13568-023-01652-6 (PMC10709273; doi:10.1186/s13568-023-01652-6)

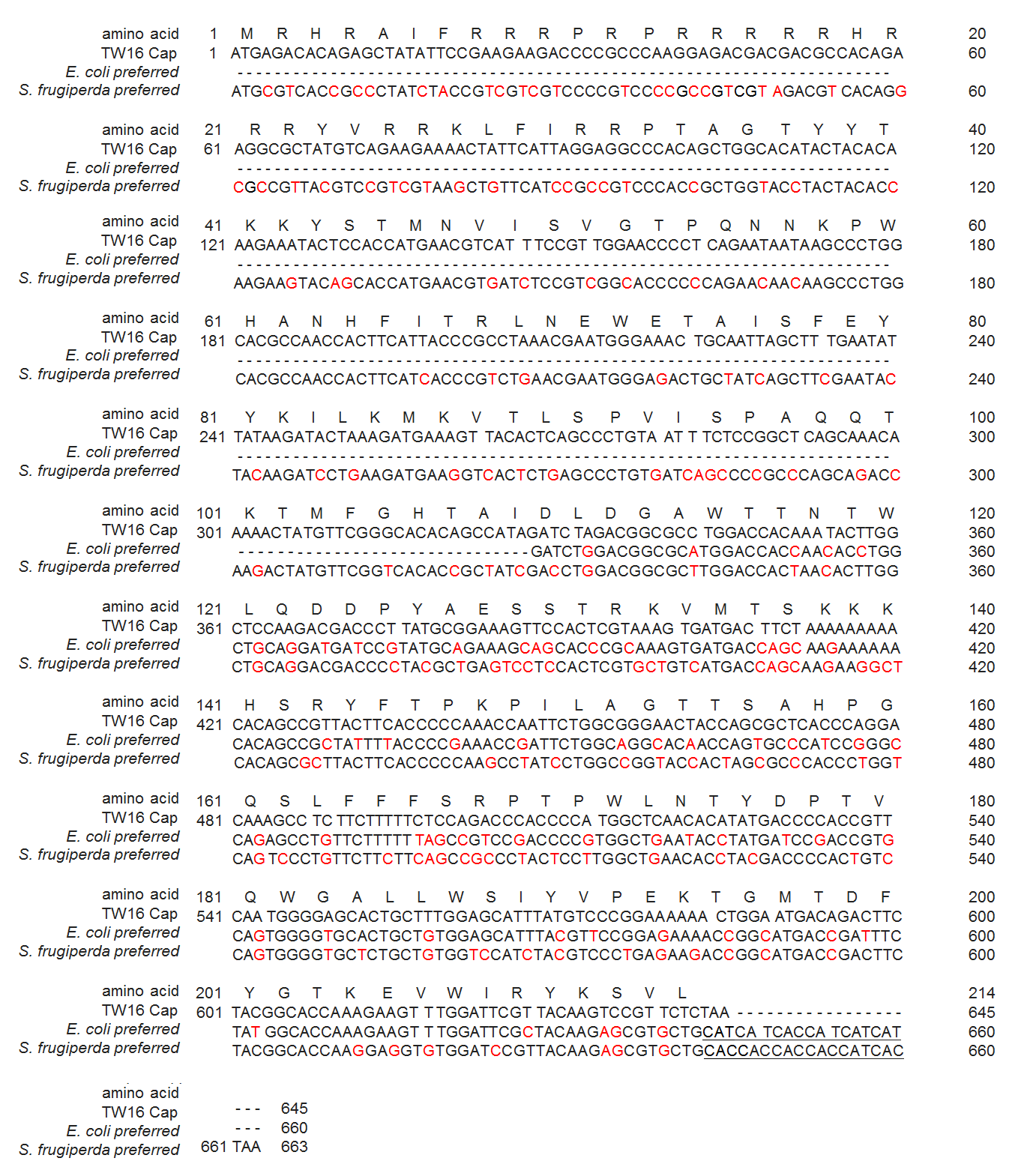

Supplement: Supplementary file 1 — Additional file 1: Fig S1 PCV3 Cap gene amino acid and nucleotide sequences. [file 13568_2023_1652_MOESM1_ESM.tif]
